# The obstacles to nurses being present with patients

**DOI:** 10.1002/nop2.723

**Published:** 2020-11-29

**Authors:** Foroozan Atashzadeh‐Shoorideh, Fatemeh Monjazabi, Ensieh Fathollahzadeh, Oujian Parastoo

**Affiliations:** ^1^ Department of Psychiatric Nursing and Management School of Nursing & Midwifery Shahid Beheshti University of Medical Sciences Tehran Iran; ^2^ Department of Medical‐Surgical Nursing School of Nursing & Midwifery Shahid Beheshti University of Medical Sciences Tehran Iran; ^3^ Flinders University College of Nursing and Health Sciences Adelaide SA Australia; ^4^ Department of Pediatric Nursing School of Nursing & Midwifery Shahid Beheshti University of Medical Sciences Tehran Iran

**Keywords:** content analysis, nurses, nursing, nursing care, obstacles

## Abstract

**Aim:**

The aim of the present research was to investigate the obstacles, which prevent nurses being present with patients.

**Background:**

It is vital for nurses to be able to spend time with patients for an accurate assessment of patients' needs to take place and to allow patients to express their concerns. The factors, which prevent nurses spending time with patients, are still unclear.

**Method:**

Data were collected using semi‐structured interviews with thirty‐five participants, including the nurses and physicians from educational hospitals of Tehran. The analysis was performed through the conventional content analysis. To achieve accuracy and trustworthiness of the data, the Lincoln and Guba criteria were used.

**Result:**

The results of the study can be summarized as: “conflict between human considerations and bureaucratic structure,” “failure to meet basic needs,” “the personal and interpersonal aspects of caring” and “safety in caring context.”

**Conclusion:**

To ensure high‐quality care, it is important to understand more fully the factors that prevent nurses spending time with patients. Interventions are needed to allow nurses to spend more time with the patients.

**Implication for nursing management:**

Health service managers should consider that the intrinsic motivation of nurses is to care for patients. They can increase the presence of nurses at patients' bedside and improve care quality by creating an attractive working environment, appreciating nurses' values, paying attention to their opinions and establishing professional communication based on mutual respect.

## 1 INTRODUCTION

Nurse presence, as a concept for scrutiny and debate, was first introduced by Sister Madeline Clemence Vaillot in 1962 (Smith, 2001). The presence of nurses with patients is an essential element of care, vital for all nursing interventions and necessary for patient safety and for performing the nursing process (Boeck, 2014). Nurse presence is an inseparable part of comprehensive care, which aims at meeting patient needs by healing the body and soul (Fahlberg & Roush, 2016). In the concept of “presence,” the sharing of human experience is an essential aspect of nursing care. In seeking to be present, nurses find a way to be with patients and be available (Kostovich et al., 2016).

## 2 BACKGROUND

The presence of nurses with patients is described as an interpersonal and intersubjective experience that changes the nurse as much as the patient. Moreover, nurse presence includes responsiveness and communication (Mohammadipour et al., 2017). The responsive behaviour of the nurse, along with respect for dignity, results in a safe and restorative environment for patients during hospitalization, helping to reassure patients and keep them safe during a vulnerable time (Bright, 2012).

The essence of nursing care is the development of an effective relationship with the patient, centred on their perceptions and needs (Mohammadipour et al., 2017). The nursing presence has been widely accepted as the core of patient–nurse communication in the nursing profession (Davis Boykins, 2014; Gardner, 1992; Riviere et al., 2019; Watson, 2009).

Nurse presence leads to an increase in nurses' mastery of patient‐related issues, beliefs and needs; and consequently, they can undertake focused activities to provide individualized and holistic care (Kostovich & Clementi, 2014). By being present with patients, nurses are able to develop trusting relationships with lead to effective interactions. Nurses are then better placed, not only to examine the physical needs of the patient, but also to pay attention to the patient's emotional needs. The nurse can meet these needs only by spending time with the patient. This allows the nurse to consider his or her body language and eye contact and makes active listening possible (Strandås & Bondas, 2018).

The expectations of the patients are sometimes different from the perceptions of the nurses, so it is good practice to seek the patient's views and to offer supportive care based on the individual's perception (White, 2014). The correct identification of needs can only be carried out when the nurse is present, encouraging the patient to express their concerns in a calm and safe environment (Stockmann et al., 2018). Meeting patient needs in terms of comfort and health depends on good clinical nursing practice, provided by paying attention to the physical, mental and spiritual needs of patients (Mojarad et al., 2019).

“Nurse Presence” has been described in multiple ways. First, according to Watson's theory, caring is an authentic, intentional, heart‐centred human presence (Pajnkihar et al., 2017). The more authentic the feelings are that the nurse conveys, the more effective the caring process will be. In fact, using this person‐centred approach assists nurses to communicate more deeply, helping patients to see themselves a valued and valuable. This approach in turn increases the sense of self‐worth and motivation among nurses (Jones, 2018).

Second, it is recommended in the Parse theory that the nurse should spend time with patients, allowing for this authentic presence. It is a special way of being with others, identifying the values and preferences of others as basic principles (Barros et al., 2017).

Nursing presence is defined by six features: uniqueness, connecting with the patient's experience, sensing, going beyond the scientific data, knowing (what will work and when to act) and being with the patient (Doona et al., 1999).

Availability can help nurses to meet the patient's emotional, physical and comfort needs, while lack of understanding and attention to needs causes fear, anxiety and worry (Weigl et al., 2016). Being in a constantly changing care environment with busy nurses, who are dealing with daily tasks, results in sense of fear and vulnerability in patients (Kieft et al., 2014). The lack of easy access to nurses can exacerbate these problems (Penque & Kearney, 2015). Patients need to feel that they are receiving care and attention (Wolf, 2017).

To achieve this goal, many concepts and theories have suggested “presence” as a central and essential concept in nursing practice. The essence of nursing care is the development of an effective relationship with the patient, centred on their perceptions and needs (Mohammadipour et al., 2017).

A review of the literature shows that despite the emphasis on the necessity and benefits of nursing presence for patients and for nurses themselves, clinical practice is often very different (Mohammadipour et al., 2017). Existing evidence shows that nursing presence depends on the culture and individual perceptions (Penque & Kearney, 2015). Various quantitative studies have examined nurse presence with patients; however, many of them offer only weak evidence due to a lack of clear definitions and biases (Anderson, 2007; Bozdoğan Yeşilot & Öz, 2017). Given the formation of concepts in the society, different perceptions of different cultures and conflicting opinions of researchers, it seems necessary to explore the nursing presence in communities so that nurses can act based on a broad vision in providing patient‐centred care while the nursing managers provide supportive strategies. Thus, a deeper exploration of this concept, focusing on the quality of communication between nurses and patients, may be an opportunity to improve the understanding of this concept. Despite numerous studies in this regard, obstacles of the being with the patient from physicians and nurses' perspective are still unclear. Besides, given the difference in the field of work of the nurses in any context, it is necessary to carry out a qualitative study to review the viewpoints of nurses to identify obstacles. The aim of the present study was to investigate the obstacles of the being with the patient by nurses based on the participants' perceptions and interpretations.

## 3 METHODS

### 3.1 Design

This is a descriptive‐exploratory study. Qualitative research has high sensitivity to describe the phenomenon, providing a comprehensive summary of events experienced by individuals (Polit & Beck, 2008).

### 3.2 Setting

To preserve the natural context (Polit & Beck, 2017), the study was conducted where the phenomenon occurred. Five educational hospitals affiliated with Shahid Beheshti University were selected. All hospitals had different wards, including internal, surgery, CCU, ICU, children, mental health and emergency department. These hospitals provide care and treatment services to more than 5 million people in Tehran. The nursing workforce at these various hospitals is almost 5,300. The numbers of beds in each of hospitals are almost 200.

### 3.3 Participants

Participants entered the study through purposive sampling (Graneheim & Lundman, 2004). Purposive sampling help to the identification and selection of information‐rich cases related to the phenomenon of interest (Palinkas et al., 2015). Thirty‐five participants, including the nurses and physicians, were selected. As nurses and physicians work tightly in wards, researchers decided to include the viewpoint of physicians in this study. Semi‐structured interviews and field notes were conducted for gathering data. The inclusion criteria included having a bachelor degree or higher in nursing for nurses, at least 1 year of clinical experience for nurses and physicians and willingness to state the experiences. The demographic characteristics of the subjects are presented in Table 1.

**TABLE 1:** The demographic characteristics of the participants

Characteristics	Number
Age (year)	
23–30	11
31–40	18
41–50	6
Gender	
Male	12
Female	23
Post	
Nurse	18
Head nurse	7
Supervisor	7
Physician	3
Work experience (year)	
01‐Oct	9
Nov‐20	23
21–30	3
Specialty	
Emergency	6
ICU	5
CCU	5
Mental health	4
Internal	7
Surgical	4
Paediatric	4
Education	
Bachelor of science	24
Master's degree	6
PhD	5

### 3.4 Data collection

After explaining the purpose of the research to the participants, semi‐structured, face‐to‐face and in‐depth interviews and field notes were conducted (Brinkmann & Kvale, 2015). The first author (faculty member of nursing with PhD degree who is familiar with qualitative research) was in charge of data collection. There was no relationship prior the study between participants and researcher. The open questions, which had open and interpretable answers, were designed as a guideline for the interview. Other follow‐up questions were asked after participants' response. The questions were as follows: “What helps you (nurse) to spend time at the patient's bedside?”, “What factors decrease the amount of time you are able to spend with patients?” The mean duration of the interviews varied between 30 min and one hour, and two participants were interviewed twice as for the first time their time was limited. The location of the interview was selected based on the participants' preference so that the interviews were performed in a hospital or places that participants felt able to relax. Data were recorded by audio tape and after each interview, as soon as possible, the researcher transcribed the interview and data were collected and analysed simultaneously until data saturation was reached (Polit & Beck, 2017).

### 3.5 Data analysis

The three‐step approach proposed by Graneheim & Lundman was used to analyse the data (Graneheim & Lundman, 2004). The participants were selected based on interview analysis and data guidance. To reach the theoretical saturation, 37 interviews were carried out with 35 participants and five field notes were conducted. Field notes allow the researcher to access the subject and record what they observe in an unobtrusive manner (Phillippi & Lauderdale, 2018). Field notes of this study made it possible for the researcher to directly observe the presence and relationship of the nurse with the patient. Then, the text of the interviews was analysed word‐by‐word, using conventional‐qualitative content analysis method (Graneheim & Lundman, 2004; Hsieh & Shannon, 2005). In this method, the data were analysed simultaneously with the data collection, so that interview was read several times, then similar texts related to the experiences of the participants were put in a text to form the analysis unit. Next, semantic units were extracted in a primary code format from the response of the participants. The extracted codes were classified based on semantic and conceptual similarity, compressed and summarized semantic units were extracted. The codes were put in sub‐categories and similar sub‐categories in a category (Graneheim & Lundman, 2004). The analysis process was performed by all authors and compared with each other, and ultimately the disagreement was resolved with the consensus. To analyse data after extraction of semantic units, MAXQDATA ver.10 software was used. From the 35th interview onwards, no new data were extracted from interviews, but the researcher carried out two further interviews to ensure data saturation.

### 3.6 Trustworthiness

Lincoln and Guba's criteria were used to examine the trustworthiness of data (Guba & Lincoln, 2004). To access valid data, the following were considered:

Allocation of sufficient time by the researcher for long‐term engagement with data, for credibility (Polit, 2010). To ensure the agreement of findings with the experiences of the participants, they were reviewed. Accordingly, the primary codes and extracted categories were shown to nurses who participated in the study to match them with their experiences for credibility (Guba & Lincoln, 2004). To observe the peer checking, two experienced and expert academics in the field of qualitative research who were not part of the research teams monitored and supervised the research process (Polit & Beck, 2012). Leaving aside judgments and preconceptions, the researcher analysed the data to avoid bias. The research team aimed to record and reported all steps of the research and the decisions made within the study to make an opportunity to follow‐up and re‐investigate the research by others. To help transferability, the way of selection, the characteristics of participants and their cultures were described. Besides, in the research report, we aimed to increase the transitivity by providing appropriate quotes (Graneheim & Lundman, 2004).

## 4 RESULTS

The results of the study led to extracting four categories including “Conflict between human considerations and bureaucratic structure,” “Failure to meet basic needs,” “Personal and interpersonal aspects of caring” and “Safety in caring context” (Table 2). The categories indicated that the experiences of nurses on the obstacles to their presence at the patients' bedside have expanded dimensions.

**TABLE 2:** The categories and sub‐categories obtaining from interviews

Categories	Sub‐categories
Conflict between human considerations and bureaucratic structure	– Documentation and bureaucracy – The conflict between the responsibility and accountability – The high workload and lack of healthcare workers
Failure to meet human basic needs	– Insufficient financial support by hospitals managers – No timely payment of salary – No fair payment system in the healthcare system – Lack of support and perceived injustice
The personal and interpersonal aspects of caring	– Personal characteristics – Inter‐ and intra‐professional communication
Safety in caring context	– The physical structure of the ward – Patients admission structure – Access to resources and equipment – Relationship with other parts of the hospitals

### 4.1 Category 1: Conflict of human considerations and bureaucratic structure

Regardless of the clinical specialism, years of work experiences and the gender of the participants, all of them stated that the first obstacle preventing the nurse presence at the patient's bedside is the conflict between human considerations and bureaucratic structure. This section includes three sub‐categories: “documentation and bureaucracy,” “the conflict between responsibility and accountability” and “high workload and lack of healthcare workers.”

#### 4.1.1 Subcategory 1‐1: Documentation and bureaucracy

All the participants stated that documentation and other written work is one of the most insurmountable obstacles to nurse presence at the patient's bedside. Writing long and detailed reports, completing patient documentation and so on are among the issues that nurses mentioned regarding this subcategory. One nurse (ICU ward) stated:

P13: In my opinion accreditation means the respectful separation of the nurse from the patient's bedside. My nurses always complete a different kind of accreditation forms and they don't have enough time for patients! (Accreditation forms are completed by nurses in clinical wards and are periodically reviewed by internal and external evaluation teams to evaluate care standards.)

One of the physicians (internist) reported:

P2: Many of the nurses in my ward are taken up with completing documentation and writing their nursing report. They write and write at the nurse's station and so spend less time with their patients.

It appears that some head nurses believe nurses should spend more time at the patient's bedside and less time on administration such as medicine and equipment requests. One nurse (emergency ward) stated that:

P10: My head nurse values the time we spend at the patient's bedside, so she gives the paper work to the secretary and sometimes she does those tasks herself.

#### 4.1.2 Subcategory 2‐1: The conflict between responsibility and accountability

The conflict between responsibility and accountability is also considered as one of the obstacles to nurse presence at the patient's bedside. Most of the nurses mentioned the conflict between their work responsibilities and feelings accountable for the work of other workers in the ward. Doing additional work such as following up the ward clerk's tasks in the case of their absence, monitoring the work of nurse assistants, training new nurses and monitoring the completion of documents and medical instructions written by new medical students are all obstacles to the nurse presence at the patient's bedside. Regarding this subcategory, a nurse (surgical ward) asserted:

P17: we are not only nurses, sometimes we are guards, secretaries, trainers, etc. we do lots of unrelated time‐consuming tasks.

#### 4.1.3 Subcategory 3‐1: The high workload and lack of healthcare workers

Long working hours, high workload, mandatory overtime, short staffing, critically ill patients and their emergency conditions are among the obstacles that were indicated by nurses. Regarding “high workload and short staffing,” one nurse (paediatric ward) emphasized:

P19: The number of the patients is very high on each shift; for example sometimes I am responsible for 10 patients on a shift and when one of them feels bad I have to take care of him or her which means other patients cannot receive enough attention.

In this regard, one of the surgeons stated:

P13: shortage of nurses with high workload inhibits nurses being at the patient's bedside.

### 4.2 Category 2: Failure to meet basic needs

The results showed that meeting their own physiological and emotional needs and need for safety could be considered an obstacle to the nurse presence at the patient's bedside. The category is made of four sub‐categories include of “insufficient financial support by hospitals managers,” “no timely payment of salary,” “no fair payment system in the healthcare system” and “lack of support and perceived injustice.”

#### 4.2.1 Subcategory 1‐2: Insufficient financial support by hospitals managers

The nurses stated that one of the obstacles of nurse presence is “insufficient financial support by hospitals managers.” They said that the managers did not appreciate nurses who work with dedication to provide excellent services and ensure patient satisfaction. They believed that, from the managers' perspective, it made no difference whether nurses spent less and more time at the patient's bedside. These issues led to a reduction in the motivation of the most active and dedicated nurses, leading them to work in the same way as other less patient‐cantered members of staff. Regarding this subcategory, a nurse and a physician emphasized:

P1: In terms of salary and merit pay, there is no difference between me that always available on the patient's bedside and a person who only sits on her chair. Merit pay should be paid to nurses who try hard to provide the best services for patients, not for every nurse.

P5: There is no difference between a nurse who always available at the patient's bedside and a person who not available. Unfortunately, sometimes that person is more appreciated‼.

#### 4.2.2 Subcategory 2‐2: No timely payment of salary

Nurses also stated that “delays in payment of salary” were among the obstacles to nurse presence at the patient's bedside.

One physician said:

P3: I saw one of nurses angered by a delay in the payment of her salary. This made her anxious so she spent less time at the patient's bedside.

#### 4.2.3 Subcategory 3‐2: No fair payment system in the healthcare system

The participants believed that a lack of fair payment system in the healthcare system and the under‐valuing of the nursing profession resulted in a reduction of nurse presence at the patients' bedside. Regarding two sub‐categories, a nurse (CCU ward) stated:

P4: I work 18 hr shifts to help the hospital run smoothly, but rather than being appreciated for this I receive the same pay as my other colleagues who have not worked those hours and I am even blamed for tasks which have not been done.

#### 4.2.4 Subcategory 4‐2: Lack of support and perceived injustice

Some nurses mentioned lack of support and perceived injustice as obstacles of nurse presence at the patient's bedside. From the nurses' perspective, the under‐valuing of nurses as important and effective members of the hospital, in comparison with physicians who are more highly valued than nurses, can discourage nurses from engaging in their careers and prevent the nurse presence at the patient's bedside. Accordingly, the subcategory “a lack of fair payment system in the healthcare system” one of the nurses stated:

P19: No one sees that I work all night and visit the patients several times, while the doctor sleeps in his room and get a higher salary than I do. Nurses have no support, for example who will support me if I am kicked by patient's relative like my coworker?!

### 4.3 Category 3: The personal and interpersonal aspects of caring

This category is made of two sub‐categories including “personal characteristics” and “inter‐professional communication.”

#### 4.3.1 Subcategory 1‐3: Personal characteristics

The nurses considered “personal characteristics” as one of the obstacles of nurse presence on patient's bedside. The motivation of the nurses, their personal, physical and family problems, the concerns of the workplace affect the quality of care service and the nurse presence at the patient's bedside. A nurse with physical, personal and the family problems cannot always concentrate on providing care; in fact, this person needs care themselves and is not able to be present at the patient's bedside. Accordingly, one of the nurses stated:

P6: when I have backache I have to sit or use my sick leave; due to the lack of personnel I cannot use it and I always feel bad so I can only do the most vital things related to the patient.

#### 4.3.2 Subcategory 2‐3: Inter‐ and intra‐professional communication

Also considered as one of the most important and effective obstacles in nurse presence at the patient's bedside. Some of the nurses indicated that lack of interest in the nursing profession and nurse manager characteristics being obstacles to nurse presence at patient's bedside. In shift work, disinterested and unmotivated nurses result in poor relationships between co‐workers. They present at the patient's bedside less often, so other nurses are left to perform the tasks, after a while, this issue leads to tiredness and indifference of the part of the more motivated and interested nurses. Regarding the subcategory, “Inter‐professional communication,” one nurse and one physician working in internal ward asserted:

P7: Working with interested colleagues is very important. I have a friend who is excited about her job and we do all the tasks without any problems. On the other hand, I had a coworker who did not love her job and postponed her tasks and I had to do her work, so I could not fulfill my own responsibilities as well as I had previously.

P8: God forbid having an un‐motivated coworker. Such a person mocks you. One of my coworkers asked me what I found to do in the patient's room, saying that I was always there.

### 4.4 Category 4: Safety in caring context

This category is made of four sub‐categories including “the physical structure of the ward,” “patient's admission structure,” “access to resources and equipment” and “relationship with other parts of the hospitals.”

#### 4.4.1 Subcategory 1‐4: The physical structure of the ward

The physical structure of the ward relates to the characteristics of the wards such as the number of occupied beds, the physical shape of the ward, the layout of the equipment, where the nurses are working there. The high patient turnover rate led to a reduction in the nurses' concentration and presence at the patients' bedside. A nurse working in the surgery department said:

P20: When I admit the patients and make her/him ready for surgery room, I deal with administrative work and I have no time for being present at the patient's bedside. I think another person should do these processes.

The physical shape of the ward is also a factor in nurse presence with patients. Where the treatment room is far from patient's rooms, nurses spend a lot of time on their feet. One of the physicians said:

P18: the structure and ergonomics of our department is not up to standard for working on it.

Subcategory 2‐4: Patient admission structure “Patient admission structure” was another effective subcategory related to the presence of nurses with patients. High turnover of patients onwards can cause an interruption or pause in the presence of nurses with patients. Regarding this subcategory, one nurse asserted:

P12: Our condition is not as same as the intensive care unit, where the patients are fixed and nurses are allocated to the same patients every day. In our unit, patients are always changing and I have to make the patients report immediately and follow up their drugs etc. I know the beds are limited and the number of patients is high.

#### 4.4.2 Subcategory 3‐4: Access to resources and equipment

Another dimension effecting nurse presence was “access to resources and equipment.” The supportive structure means the interactions of other administrative and non‐administrative departments of the hospital with any of the treatment departments is necessary. Most of the equipment and medicine needs to be ordered and delivered to the ward. Sometimes providing the equipment and medicines is a time‐consuming process, which prevents nurse from being present at the patient's bedside. Sometimes the time taken to track the purchase of equipment and medicine is greater than the time spent on care itself and presence at the patient's bedside. A head nurse said:

P14: If the required equipment is not available in the ward I have to follow it up several times. The drug ordering system is better, you only need to call the pharmacy and the answer is yes or no.

#### 4.4.3 Subcategory 4‐4: Relationship with other parts of the hospitals

Lack of computer systems in each ward and the high number of drugs and equipment requests was another time‐consuming factor. All the nurses of a ward need to send their request to pharmacy or medical equipment department in a limited time period, so it consumes the nurses time, which prevents them from being present at the patient's bedside. Regarding this subcategory, a nurse asserted:

P16: One ward with 40 patients and only 1 computer system! I have to wait to complete the medicine and equipment requests, to follow up test results, requesting CT scan image, etc.

## 5 DISCUSSION

The results of the present study showed that the obstacles to being with patients included a conflict between human considerations and bureaucratic structure, failure to meet basic needs, the personal and interpersonal aspects of caring and safety in caring context. In addition, the participants believed that there were many issues related to each nurse and their ward that prevented them from being with the patients.

Conflict of human considerations and bureaucratic structure is as one of the important issues relating to the presence of the nurse with patients. Based on the participants' perspective, the most important obstacle of nurse presence on patient's bedside was that managers' prioritize the completion paperwork over nurses spending time with patients. The results of other studies indicated the high rate of time‐consuming reporting and documentation by nurses, which prevents them from giving care and offering their presence at the patient's bedside (Nursalam et al., 2018; Ommaya et al., 2018).

The conflict between direct patient care and other responsibilities is another factor that was mentioned as an obstacle to nurse presence. Giving the specific responsibility to each nurse, for example, a nurse responsible for educating novice nurses, or a nurse responsible for requesting equipment can help other nurses to provide better services (Duffield et al., 2016). Most of the nurses mentioned the high workload and staff shortage as one of the most important factors preventing their presence with patients. In Mirzaei research reported that nurses believed that staff shortages in different wards of the hospitals led to overwork and impossible demands; however, the income of nurses is not commensurate with their workload (Mirzaei et al., 2018).

Other studies also mentioned that short staffing and heavy workload affected the physical health of the nurses. Problems arose such as musculoskeletal pain, chronic low back pain, chronic stress, job dissatisfaction, anxiety, depression and career exhaustion (Greaves et al., 2018; Lu et al., 2019; Risman et al., 2016; Shieh et al., 2016). This also led to a reduction in patients' satisfaction with the care they received, a poor connection between nurse and patient, risks to the safety of patients, a reduction in responsiveness to patient' needs, including emotional, mental, spiritual needs, as well as increase in possible errors and hospital infections (Aiken et al., 2018; Lu et al., 2019; Weigl et al., 2016).

The financial and basic needs of the nurses are ignored by managers, and this issue affects the efficiency of the nurses. The results of a study performed in Taiwan revealed that an increase in working hours and workload of the nurses and no increase in their salary has an impact on the quality of care (Chang & Hsiu‐Hui, 2019). The results of Liu's review study showed that the main attributes of job satisfaction in nurses are fulfilment of desired needs in the work settings: good working conditions and job value or equity. These attributes are influenced by antecedent conditions like demographic, emotional state, work characteristics and environmental variables. Additionally, the consequences of nurses' job satisfaction have a significant impact on both nurses and patients (Liu et al., 2016). Many Iranian nurses are not satisfied with their work due to an overwhelming workload, insufficient time and inadequate resources (Valizadeh et al., 2016), inappropriate work conditions, lack of support and discrimination in payments (Aloustani et al., 2020).

The personal and interpersonal aspects of care are the third category of evidence obtained in the present research. Considering the nurse as a person with basic needs, aches and pains, financial and family problems, as well as paying attention to nurses' leave requests and changing their ward are among the factors influencing the nurse presence. Besides, the selection of interested people in this job and career in university and hospitals can reduce interpersonal problems (Chen & Lucas, 2017; Nepangue‐Seaman et al., 2016). Most of the nurses with intrinsic and individual motivation to care for others work without considering their payment and organization climate (Sodeify, 2018).

Safety in the caring context is the last category in the present research. Safety could be considered as a facilitator of the being with the patient. Donabedian believed that organization structure affected the care process (Donabedian, 1988). Factors such as the number and composition of employees, the available resources and their organization affect clinical activity (Donabedian, 1988). Safety in healthcare settings is a multidimensional construct involving factors such as patient safety, occupational safety and quality improvement, all designed to protect hospital staff and the public (Muir‐Cochrane & James, 2020). The physical structure of most of the medical University hospitals is old and has not changed alongside many equipment changes, the extent of the diseases and the number of patients. Studies have shown that severe shortages of nursing staff, changing patients' needs and problems with treating and caring costs, lead to major challenges in designing and delivering services to patients. Flexible design, adequate equipment and access to resources in the ward by nurses can improve nurses' performance and patient satisfaction (Gallant & Lanning, 2001).

## 6 CONCLUSION

Being present with the patient is one of the main roles of the nurse. Various issues have had an impact on the ability and willingness of nurses to spend time with patients. Recognition of these issues could lead to changes in various areas: hospital managers need to give attention to the payment of nursing staff; an improvement in documentation systems could create conditions for the nurse to be present at the patient's bed such as using an audio reporting system, improve the hospital's physical structures.

### 6.1 Strengths and weaknesses of the study

Although the participants shared many of their experiences, the study still has limitations. The present study was conducted with nurses and physicians, who were attended to Shahid Beheshti University of Medical Sciences Hospitals so findings should be used carefully with other settings; on the other hand, the perspective of patients about nurses' presence at their bedside will be different. It is suggested to perform a similar study with patients.

### 6.2 Implication for nursing management

To improve the quality of nursing services and increase their presence at the bedside, the obstacles should be explored and evaluated. The results indicated that nurses have intrinsic motivations to be present at the patient's bedside. Thus, managers can improve the quality of the service by creating an attractive workplace, appreciating their nurses, respecting their comments, creating a professional connection based on mutual respect, considering the mental and emotional needs of the nurses, prioritizing job satisfaction, increasing sense of commitment and accountability, addressing financial issues and fairness in the nursing system.

## CONFLICT OF INTEREST

The authors did not declare any conflict of interest.

## ETHICAL APPROVAL

This study was approved by Committee of Ethics in Human Research at Shahid Beheshti Medical University (IR.SBMU.RETECH.REC.1397.614). To perform the interviews, research aims and procedures were explained to the participants and informed written consent for voice recording and observing the caring nurses for field notes and using the data was obtained from each participant (nurses and physicians). The time and location of the interviews were chosen according to the participants' convenience. The audio file of the interview and the participants' information were kept confidential by the researcher.

## Data Availability

The corresponding author will provide the data of the article upon request.
